# Induction of Apoptosis in Thymocytes by Prostaglandin E_2_ In Vivo

**DOI:** 10.1155/1992/80863

**Published:** 1992

**Authors:** Antonio Mastino, Mauro Piacentini, Sandro Grelli, Cartesio Favalli, Francesco Autuori, Lucio Tentori, Serafina Oliverio, Enrico Garach

**Affiliations:** 1 Department of Experimental Medicine and Biochemical Science University of Rome “Tor Vergata” Via O. Raimondo Rome 00173 Italy; 2 Department of Biology University of Rome “Tor Vergata” Via O. Raimondo Rome 00173 Italy; 3 Institute of Experimental Medicine C.N.R Viale Marx 15 Rome 00156 Italy

**Keywords:** Prostaglandin E_2, apoptosis, thymocyte subsets, T-cell development, tissue transglutaminase

## Abstract

In vivo administration in mice of a synthetic analog of prostaglandin E_2_ (PGE_2_) caused a selective and dramatic decrease of CD4^+^CD8^+^ double-positive, CD3/T-cell-receptor-*αb*^10^ cells in the thymus. This loss was corticosteroid-independent and not affected by Cyclosporin A. The disappearance of CD4^+^CD8^+^ thymocytes was strictly correlated with the induction of apoptosis inside the thymus as shown by morphological studies and by the induction of intracellular transglutaminase expression. Considering that PGE_2_ has been found to be produced by different cell populations inside the thymus, these results indicate that PGE_2_ may act as endogenous signals for apoptosis during T-cell differentiation.


*Developmental Immunology*, 1992, Vol. 2, pp. 263–271
Reprints available directly from the publisher
Photocopying permitted by license only

© 1992 Harwood Academic Publishers GmbH
Printed in the United Kingdom

### Induction of Apoptosis in Thymocytes by Prostaglandin $E_2$ In Vivo

ANTONIO MASTINO,*† MAURO PIACENTINI,‡ SANDRO GRELLI,†§ CARTESIO FAVALLI,† FRANCESCO AUTUORI,‡ LUCIO TENTORI,† SERAFINA OLIVERIO,‡ and ENRICO GARACI†

*†Department of Experimental Medicine and Biochemical Science, University of Rome "Tor Vergata," Via O. Raimondo, 00173 Rome, Italy*
*‡Department of Biology, University of Rome "Tor Vergata," Via O. Raimondo, 00173 Rome, Italy*
*§Institute of Experimental Medicine C.N.R, Viale Marx 15, 00156 Rome, Italy*

In vivo administration in mice of a synthetic analog of prostaglandin $E_2$ ($PGE_2$) caused a selective and dramatic decrease of $CD4^+CD8^+$ double-positive, CD3/T-cell-receptor-$\alpha b^{lo}$ cells in the thymus. This loss was corticosteroid-independent and not affected by Cyclosporin A. The disappearance of $CD4^+CD8^+$ thymocytes was strictly correlated with the induction of apoptosis inside the thymus as shown by morphological studies and by the induction of intracellular transglutaminase expression. Considering that $PGE_2$ has been found to be produced by different cell populations inside the thymus, these results indicate that $PGE_2$ may act as endogenous signals for apoptosis during T-cell differentiation.

KEYWORDS: Prostaglandin $E_2$, apoptosis, thymocyte subsets, T-cell development, tissue transglutaminase.

### INTRODUCTION

It is generally acknowledged that prostaglandin $E_2$ ($PGE_2$) exerts a powerful modulatory action on mature T cells (Goodwin, 1985; Vercammen and Ceuppens, 1987; Betz and Fox, 1991). Conversely, little information exists on its effects in the course of T-cell differentiation (Rinaldi-Garaci et al., 1983). The programmed cell death (PCD) of thymocytes is considered to be a crucial event that occurs during the intrathymic phase of T-cell differentiation. This death of physiological significance occurs in tissues by an active cellular phenomenon of self-destruction, called "apoptosis." It requires coordinate expression of regulatory proteins, as bc12, and enzymes, as $Ca^{++}/Mg^{++}$-dependent endonuclease and tissue transglutaminase (tTG), causing morphological modifications of the cell and leading to a final irreversible damage of DNA, characterized by molecule fragmentation (Wyllie et al., 1980; Arends and Wyllie, 1991; Piacentini et al., 1991a, 1991b). Apoptosis would lead to the controlled removal of thymocytes, during both positive and negative selection of functionally mature T cells (Rothemberg, 1990; Boyd and Hugo, 1991; von Boehmer, 1991; Zugic, 1991). The intrinsic mechanisms and the biochemical mediators of PCD in thymocytes in vivo are not yet clear (McConkey et al., 1990b). Moreover it is difficult to understand how T-cell-receptor (TCR)-mediated signals could result in two distinct thymocyte fates, that is, protection from PCD in the case of positive selection or induction of PCD in the case of negative selection. Recently, it has been demonstrated that an intracellular increase of cAMP stimulates PCD in rat and mouse thymocytes in vitro (McConkey et al., 1990a; Suzuki et al., 1991). Here we report the evidence that administration in mice of a synthetic analog of $PGE_2$, 16,16-dimethyl-$PGE_2$ methyl ester (DI-M-$PGE_2$), induces apoptosis of thymocytes in vivo, and that thymocytes at phenotypically different stages of differentiation show variable sensitivity to $PGE_2$.

*Corresponding author.

### RESULTS

### Effects of $PGE_2$ Administration on Phenotypically Identified Thymocyte Subsets

DI-M-$PGE_2$, a long-acting synthetic analog of

$PGE_2$, was administered i.p. into adult C57BL/6 mice at different doses in a single daily injection for a time ranging from 1 to 8 consecutive days. Thymuses were then collected and thymocytes were analyzed by flow cytometry using specific monoclonal antibodies. Results showed a dramatic and selective depletion of $CD4^+CD8^+$ cells. The effect was directly dependent on the dose, the number of the injections, and the time after the administration, with a maximum reached 12 hr after the last of four injections at the dose of 1 mg/kg/day (Figs. 1A to 1D). The prolongation of the treatment did not lead to further modifications. Regarding the expression of CD3 and TCR-$\alpha\beta$ molecules, thymocytes can be subdivided into negative cells and into low (lo), intermediate (int), and high (hi) expressing cells, corresponding to successive stages of T-cell differentiation (Ohashi et al., 1990). Dose-effect experiments demonstrated that CD3/TCR-$\alpha\beta^{lo}$ cells were the most sensitive to DI-M-$PGE_2$ action, followed by CD3/TCR-$\alpha\beta^{int}$, and negative cells (Figs. 1E to 1L). Conversely, CD3/TCR-$\alpha\beta^{hi}$, $CD4^+$, or $CD8^+$ single-positive thymocytes, expressing a "mature" phenotype, were highly resistant to DI-M-$PGE_2$. Looking at absolute numbers of thymocytes, a fall in all subsets in mice treated with DI-M-$PGE_2$ was observed, with the most dramatic change noted in the $CD4^+CD8^+$ cells. This was related to a decrease of thymus total cellularity after $PGE_2$ treatment (control diluent=$1.0\times10^8\pm0.1\times10^8$ cells, mean±SD, $n$=6; DI-M-$PGE_2$=$1.6\times10^7\pm2.0\times10^6$ cells, $n$=3, dosage= 1 mg/kg/day×4). The effect was reversible, as demonstrated by a good and progressive recovery of all subsets, which followed stopping treatment.

### Effect of $PGE_2$ Administration on Tissue Transglutaminase Levels and on Morphological Features of Apoptosis in the Thymus

No evidence for a migration of $CD4^+CD8^+$ cells to peripheral lymphoid organs, after $PGE_2$ administration, as detected by flow cytometry at spleen and lymphonode level, was observed. We have then investigated if the disappearance of $CD4^+CD8^+$, CD3/TCR-$\alpha\beta^{lo}$, and thymocytes after DI-M-$PGE_2$ administration could be related to the intrathymic PCD of thymocytes that naturally occurs, particularly in cortical $CD4^+CD8^+$ thymocytes, during T-cell differentiation. Recently, in vitro and in vivo experiments have clearly demonstrated that apoptotic cells, both of normal and neoplastic origin, specifically express high levels of tTG (Fesus et al., 1987, 1989; Arends and Wyllie, 1991; Piacentini et al., 1991a, 1991b). By contrast, the enzyme expression is not enhanced during necrosis (Fesus et al., 1987). tTG, by catalizing covalent cross links between polypeptide chains, leads to the assembly of a stable protein scaffold (insoluble in detergents and chaotropic agents) that prevents the release of harmful molecules from the dying cell before its final degradation by phagocitosis (Fesus et al., 1989). The induction of tTG could so be considered as an early event during apoptosis. On the basis of these findings, we monitored the expression of tTG, in parallel with the morphology of thymus cells, in order to characterize apoptosis after DI-M-$PGE_2$ in vivo administration. Upon a single DI-M-$PGE_2$ injection, tTG activity was increased over the controls as early as 3 hr, reaching a two-fold increase at 24 hr. Repeated treatments had additive effects in enhancing the enzyme activity (Table 1). The effect of $PGE_2$ was limited to the thymus, as indicated by the absence of induction of tTG in other organs, such as spleen (control= 0.75±0.04 nmol/hr/mg protein, mean±SD, $n$=3; DI-M-$PGE_2$=0.35±0.06 nmol/hr/mg protein, $n$=3) or liver (control=0.62±0.05 nmol/hr/mg protein, $n$=3; DI-M-$PGE_2$=0.45±0.08 nmol/hr/mg protein, $n$=3). Immunohistochemical analysis of thymus

TABLE 1
Tissue Transglutaminase (tTG) Activity of Thymuses from DI-M-$PGE_2$ Treated Mice[a]

| Treatment | | tTG activity (% of control) |
|---|---|---|
| No. of injections (once a day) | Time after last injection (hr) | |
| 1 | 3 | 150±12 |
| 1 | 12 | 183±41 |
| 1 | 24 | 198±35 |
| 1 | 48 | 161±30 |
| 1 | 72 | 155±24 |
| 2 | 3 | 298±52 |
| 3 | 3 | 315±48 |
| 4 | 3 | 277±36 |
| 2 | 24 | 212±37 |
| 3 | 24 | 225±45 |
| 4 | 24 | 214±30 |

[a]Transglutaminase activity was measured by detecting the incorporation of ($^3$H)putrescine into N,N'-dimethylcasein and calculated as nanomoles of ($^3$H)putrescine incorporated into protein per hour, expressed as a percentage from values obtained in mice treated with control diluent (0.24±0.08 nmol/hr/mg protein). Data are the cumulative means±SD of triplicate determinations of each individual thymus, derived from five different experiments (total $n$=30 for each experimental group).

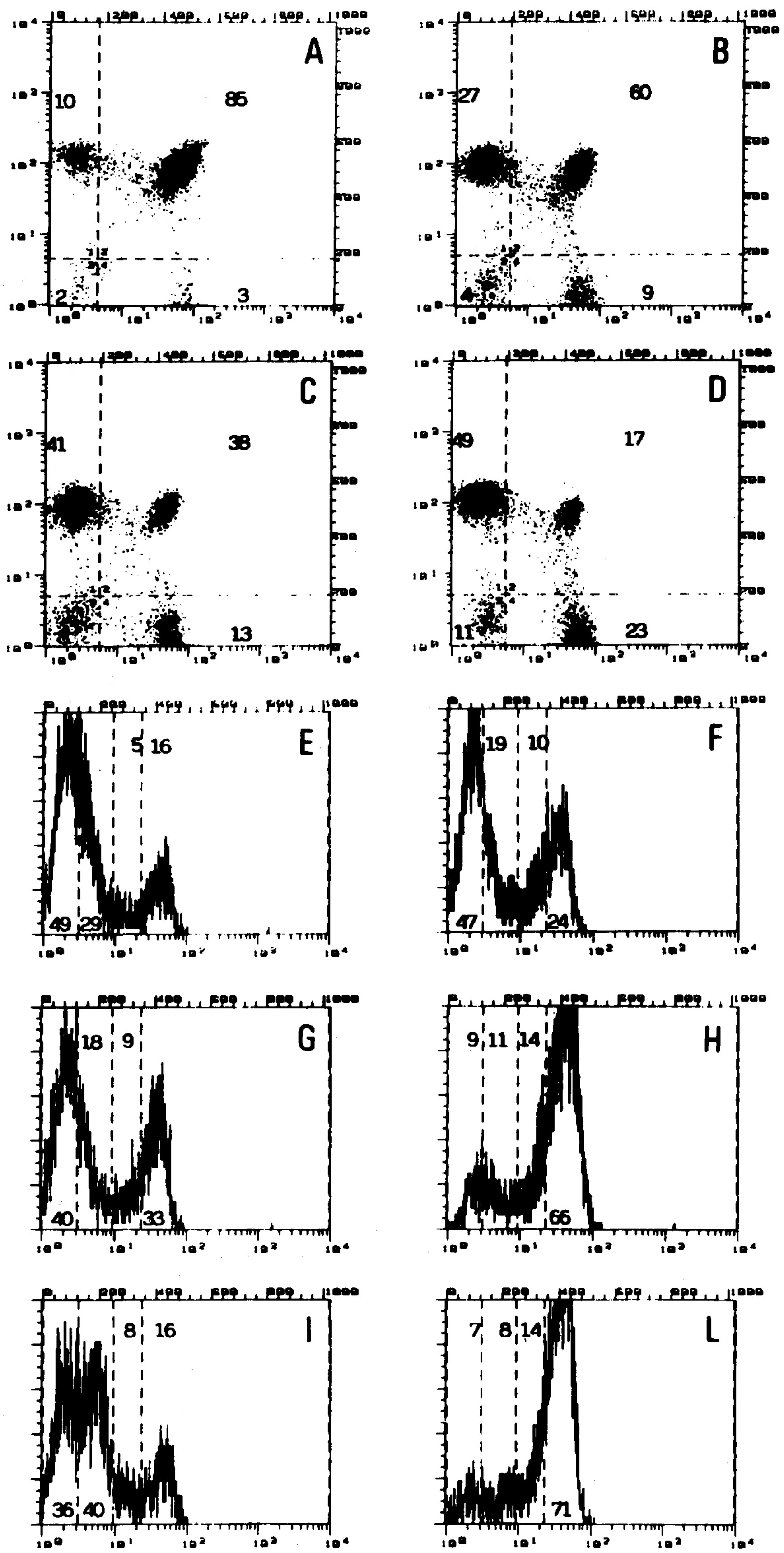

FIGURE 1. $PGE_2$ in vivo administration causes the selective loss of $CD4^+CD8^+$ and CD3/TCR-$\alpha\beta^{lo}$ thymocytes. C57BL/6NCr BR male mice were injected i.p. with control diluent (A, E, I) or 16,16-dimethyl prostaglandin $E_2$ (Di-M-$PGE_2$) at doses of 0.25 mg/kg (B and F), 0.5 mg/kg (C and G), and 1 mg/kg (D, H, and L) once a day for 4 consecutive days. Immunofluorescence staining and flow cytometry analysis were performed 12 hr after the last injection. The following antibodies were utilized: phycoerythrin conjugate antimouse L3T4 and fluorescein conjugate antimouse Lyt-2 for a two-color analysis of CD4 (vertical axis) and CD8 (horizontal axis) positive cells, respectively (A to D); fluorescein conjugate anti-CD3- for a single-color analysis (E to H); fluorescein conjugate anti-$\alpha\beta$ TCR (H57-597 mAb) for a single-color analysis (I and L). In single-color analysis, cell numbers (same full scale) are represented in the vertical axis and fluorescence, on the logarithmic scale, was plotted on the horizontal axis. The dashed lines in two-color analysis (A to D) indicate quadrant boundaries obtained by limiting 99.8% of the background events in the lower-left quadrant. The dashed lines in single-color analysis (E to L) indicate the upper and lower boundaries of CD3/TCR-$\alpha\beta$ lo, int, or hi populations for comparison among treatment groups. The first boundary was obtained by limiting 99.7% of the background events, and the others were set arbitrarily on the basis of the curve profile obtained in control samples. Numbers in the cytographs indicate percentages of cells within markers. The experiment was repeated six (A to D) and three (E to L) times, using three mice for each experimental group, with similar results within groups.

TABLE 2
Effect of $PGE_2$ Administration on CD4- and CD8-Identified Thymocytes in Adrenalectomized Mice[a]

| Group | Treatment | Percent of total (mean±SD) | | | |
|---|---|---|---|---|---|
| | | CD4$^-$CD8$^-$ | CD4$^+$CD8$^+$ | CD4$^+$CD8$^-$ | CD4$^-$CD8$^+$ |
| 1 | None | 1.6±0.6 | 83.5±1.0 | 10.0±1.0 | 4.7±0.6 |
| 2 | Di-M-$PGE_2$ | 4.3±1.2[b] | 55.6±13.0[b] | 30.9±7.3[c] | 9.2±4.5[d] |
| 3 | Adrenalectomy | 1.2±0.6[d] | 79.3±2.8[d] | 11.6±1.8[d] | 7.9±0.4[e] |
| 4 | Adrenalectomy plus Di-M-$PGE_2$ | 6.2±1.4[f,g] | 54.9±7.3[f,h] | 27.1±3.9[f,g] | 11.8±2.0[f,i] |

[a]Normal or adrenalectomized mice were injected with Di-M-$PGE_2$ at a dose of 0.25 mg/kg/day for 4 consecutive days (groups 2 and 4, respectively). Twenty-four hours after the last injection, thymuses were collected and flow cytometry analysis of thymocyte subsets was performed. Untreated (group 1) or adrenalectomized (group 3), sex, and age matched controls were also tested. Results represent percentage mean values±standard deviation obtained from three mice individually tested. Statistical analysis was performed by Student's *t*-test.
[b]$P<0.05$ against corresponding value of group 1.
[c]$P<0.01$ against corresponding value of group 1.
[d]N.S. against corresponding value of group 1.
[e]$P<0.005$ against corresponding value of group 1.
[f]N.S. against corresponding value of group 2.
[g]$P<0.005$ against corresponding value of group 3.
[h]$P<0.01$ against corresponding value of group 3.
[i]$P<0.05$ against corresponding value of group 3.

after DI-M-$PGE_2$ administration showed a large induction of tTG protein in several cells mainly localized in the thymus cortex (Figs. 2A to 2C). The morphology of positive cells showed the distinctive features of apoptosis (condensed chromatin and nuclear fragmentation) that appeared unequivocal when the immunostaining was performed in single-cell suspensions of thymocytes freshly isolated from DI-M-$PGE_2$ treated mice (Fig. 2D).

### Effect of $PGE_2$ Administration on CD4- and CD8-Identified Thymocytes in Adrenalectomized Mice

Considering that a cell loss of CD4$^+$CD8$^+$ thymocytes, similar to that observed by us after DI-M-$PGE_2$ treatment, is also induced in vivo by glucocorticoids (Screpanti et al., 1989), we have investigated if the elimination of CD4$^+$CD8$^+$ thymocytes caused by $PGE_2$ could be mediated by endogenous steroid production. In fact, it is known that glucocorticoids induce apoptosis of thymocytes (Wyllie et al., 1980; Wyllie and Morris, 1982; Ojeda et al., 1990). Therefore, we have repeated the experiments in adrenalectomized mice. After 4 days of treatment at the dose of 0.25 mg/kg/day (the maximal tolerated dose of DI-M-$PGE_2$ by adrenalectomized mice), DI-M-$PGE_2$ administration resulted in a cell loss of CD4$^+$CD8$^+$ thymocytes, with respect to untreated controls, that was similar in intact as well as in adrenalectomized mice (Table 2). Thus, it is reasonable to hypothesize that $PGE_2$ action on thymocytes is not mediated by endogenous corticosteroids.

### Effect of $PGE_2$ Administration on CD4- and CD8-Identified Thymocytes in Cyclosporin A-Treated Mice

It has been previously reported that apoptosis of thymocytes induced in vivo by anti-CD3 antibodies can be inhibited by Cyclosporin A (CsA) (Shi et al., 1989). In order to verify an eventual relationship between apoptosis induced in vivo

FIGURE 2 Immunolocalization of tTG protein in mouse thymus and freshly isolated thymocytes. Immunostaining both on paraffin included thymus sections and freshly isolated thymocytes was performed 3 hr after the last DI-M-$PGE_2$ injection using an affinity-purified monospecific rabbit IgG against soluble "tissue transglutaminase." Biotinylated goat antirabbit IgG was used as second antibody followed by a preformed avidin-horseradish peroxidase complex. Cells were counterstained in Mayer's hemalum. In thymus from control animals, the positivity to the anti-tTG antibody is limited to the endothelial cells lining the vessels (A). On the contrary, note the presence in the thymus cortex of many immunopositive (arrows) presumptive preapoptotic thymocyte after $PGE_2$ administration (B); Bar=60 $\mu$m. Higher magnification of the thymus cortex from $PGE_2$-treated animals (C); Bar=12 $\mu$m. Intense positivity to anti-tTG antibody is localized in cells showing a picknotic nucleus (arrows). Isolated thymocytes. After extensive washing in PBS, cells were smeared on slides, fixed in 2.5% paraformaldehyde, and, after immunostaining, counterstained with Mayer's hemalum. In thymocytes from control mice, no positive reaction was observed (data not shown); in cells isolated from $PGE_2$-treated animals, an increase in tTG protein expression is observed (D); Bar=60 $\mu$m. Note the intense staining of shrinked cells showing the condensed chromatin marginated at the cell periphery typical of apoptotic bodies (inset, Bar=12 $\mu$m). High concordant results were obtained in three independent experiments. (See Colour Plate VII at the back of this publication).

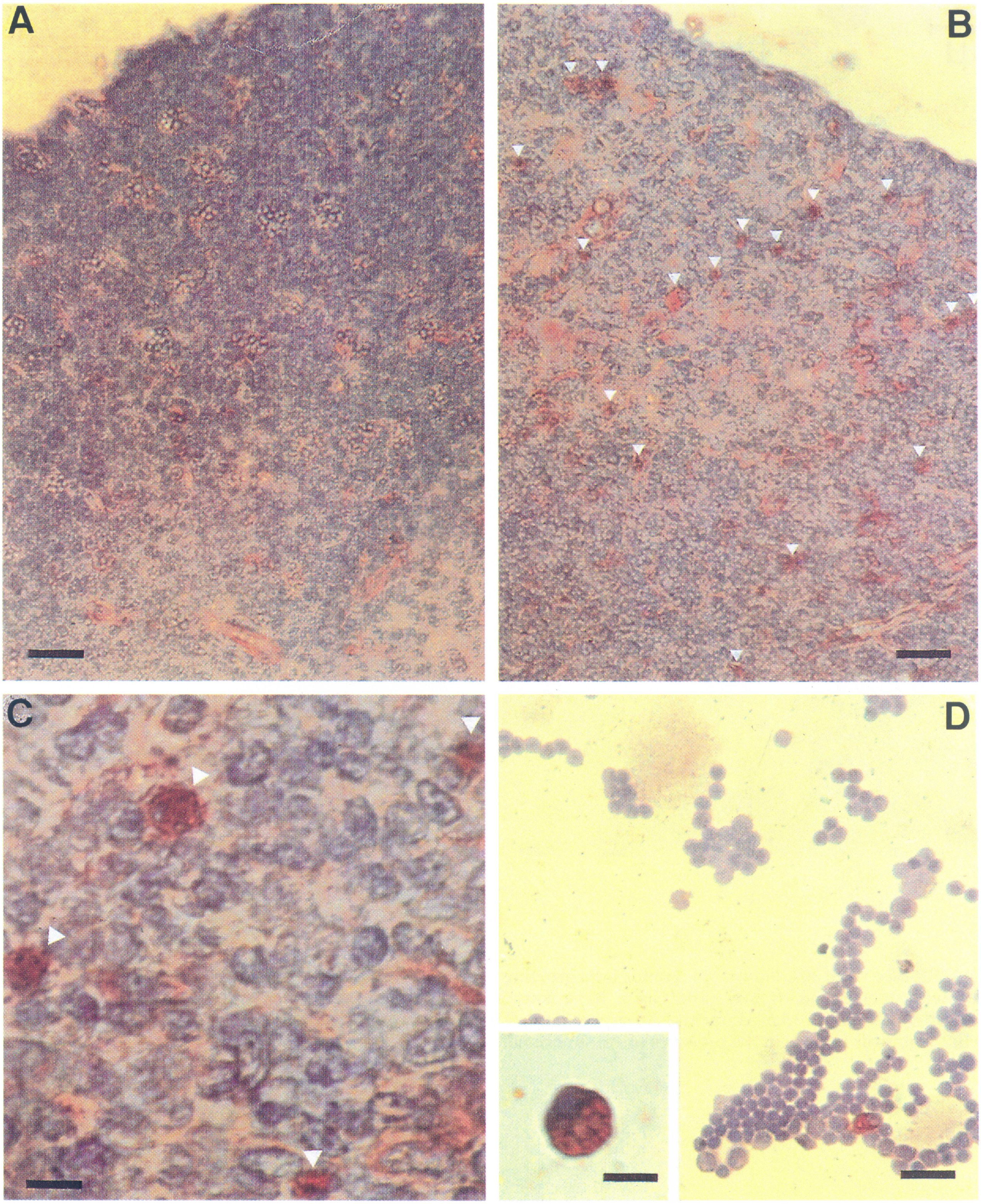

FIGURE 2.

by anti-CD3 antibodies and that induced by DI-M-$PGE_2$, we have investigated the effects of a simultaneous administration of $PGE_2$ and CsA. The flow cytometry analysis performed 24 hr after the last treatment did not show any difference between the cell loss of $CD4^+CD8^+$ thymocytes in mice treated with DI-M-$PGE_2$ alone and those treated with DI-M-$PGE_2$ plus CsA (Table 3). In addition, no changes in tTG activity were observed (data not shown).

### DISCUSSION

A restricted number of experimental models of induction of thymocyte apoptosis has so far been described. In fact, apoptosis of thymocytes has been unequivocally proved to be induced in vitro by glucocorticoids (Wyllie et al., 1980; Wyllie and Morris, 1982; Ojeda et al., 1990), radiations (Yamada and Ohyama, 1988), calcium ionophores and phorbol ester (Kizaki et al., 1989), or in vitro as well as in vivo by anti-CD3 antibodies (Shi et al., 1989; Smith et al., 1989) and some peptide antigens (Jenkinson et al., 1989; Murphy et al., 1990). Our results provide the first direct evidence for a pharmacological induction of thymocyte apoptosis in vivo. In fact, it is described by a simple and highly reproducible pharmacological experimental model of thymocyte apoptosis in vivo. Moreover, the recently obtained evidence for the induction of thymocyte apoptosis in vitro by $PGE_2$ and other intracellular cAMP elevating agents (McConkey et al., 1990a; Suzuki et al., 1991) furnishes a clear in vitro experimental support to the observation we have obtained in vivo. The in vivo $PGE_2$-induced PCD includes pecularities that extend and clarify the previously described thymocyte apoptosis features. In fact, it is not mediated by endogenous glucocorticoids, nor inhibited by CsA as in vivo CD3-induced PCD. Indeed, glucocorticoids seem not to be produced inside the thymus, nor could radiations or anti-CD3 antibodies constitute physiological triggers for thymocyte apoptosis. On the contrary, $PGE_2$ are produced in large amounts inside the thymus by a wide variety of cell types as thymic epithelial cells, dendritic cells, macrophages (Gallily et al., 1985; Homo-Delarche, 1985; Nieburgs et al., 1985), and also nurse cells as recently reported (McCormack et al., 1991). Interestingly, a selective elimination of double-positive immature thymocytes by a thymic epithelial cell line has been reported in vitro (Nakashima et al., 1990); on the other hand, it has been recently demonstrated that thymic macrophages or dendritic cells are associated to different subsets of developing thymocytes, suggesting a role for thymic rosettes, consisting of thymocytes attached to a central stromal cell, in the maturation steps in the thymic cortex (Shortman and Vremec, 1991). Thus, it is possible that $PGE_2$, endogenously produced by stromal cells inside the thymus during the intercellular contacts, which seem to play an important role during T-cell development, could locally reach concentrations active to induce cell death of thymocytes by apoptosis. The different sensitivity of CD3/TCR-$\alpha\beta^{lo}$, CD3/TCR-$\alpha\beta^{int}$, or CD3/TCR-$\alpha\beta^{hi}$, thymocytes to $PGE_2$, could be involved in

TABLE 3
Effect of $PGE_2$ ADministration on CD4- and CD8-Identified Thymocytes in Cyclosporin-A-Treated Mice[a]

| Group | Treatment | Percent of total (mean±SD) | | | |
|---|---|---|---|---|---|
| | | $CD4^-CD8^-$ | $CD4^+CD8^+$ | $CD4^+CD8^-$ | $CD4^-CD8^+$ |
| 1 | None | 1.2±0.8 | 87.7±1.1 | 13.1±1.0 | 4.0±0.7 |
| 2 | Di-M-$PGE_2$ | 3.9±1.2[b] | 39.6±9.7[c] | 37.1±9.7[d] | 19.4±4.1[c] |
| 3 | Cyclosporin A | 1.4±0.4[e] | 82.8±2.2[e] | 11.4±1.8[e] | 4.4±0.6[e] |
| 4 | Cyclosporin A plus Di-M-$PGE_2$ | 6.1±1.6[f,g] | 41.6±6.9[f,h] | 32.2±2.8[f,h] | 20.1±2.5[f,h] |

[a]Mice were injected with: Di-M-$PGE_2$ at a dose of 0.5 mg/kg/day for 4 consecutive days (group 2); Cyclosporin A at a dose of 50 mg/kg/day for 4 consecutive days (group 3); both Di-M-$PGE_2$ plus CsA (group 4). Twenty-four hours after the last injection, thymuses were collected and flow cytometry analysis of thymocyte subsets was performed. A group of sex- and age-matched controls was also tested (group 1). Results represent percentage mean values±standard deviation obtained from three mice individually tested. Statistical analysis was performed by Student's *t*-test.
[b]$P<0.05$ against corresponding value of group 1.
[c]$P<0.005$ against corresponding value of group 1.
[d]$P<0.001$ against corresponding value of group 1.
[e]N.S. against corresponding value of group 1.
[f]N.S. against corresponding value of group 2.
[g]$P<0.01$ against corresponding value of group 3.
[h]$P<0.001$ against corresponding value of group 3.

the mechanism that leads to opposite final events (selection or removal by PCD) in different phases of T-cell differentiation. Our results could contribute to explain the mechanism involved in the physiological elimination of the majority of thymocytes at the $CD4^+CD8^+$ stage. A very rapid clearance by macrophages of apoptotic thymocytes could result in the difficulty to identify well the physiologically occurring phenomenon. On the other hand, the effects caused by $PGE_2$, at the pharmacological doses we have used, could overcome the capacity of phagocytic cells to eliminate dying cells, thus rendering the phenomenon as evident when using an early marker of apoptosis as tTG.

### MATERIALS AND METHODS

### Animals

Male C57BL/6NCrBR, 4-week-old mice, purchased from Charles River Italia (Como, Italy), and male C57BL/6J, 6-week-old adrenalectomized or sham-adrenalectomized mice, purchased from Nossan (Milan, Italy), were used.

### Drugs and Treatment

*Prostaglandin*

Mice were injected i.p. with 16,16-dimethyl prostaglandin $E_2$ (Di-M-$PGE_2$) (Cayman Chem. Co., Ann Arbor, MI) at the doses of 0.25 mg/kg body weight, 0.5 mg/kg, and 1 mg/kg once a day for a time ranging from 1 to 4 consecutive days.

### Cyclosporin A

CsA (Sandoz, Basel, Switzerland) was administered at the dose of 50 mg/kg of body weight, daily for 4 consecutive days, alone or immediately after Di-M-$PGE_2$ administration.

### Immunofluorescence Staining and Flow Cytometry Analysis

Thymuses were individually processed 12 hr after the last injection by gentle teasing in RPMI 1640. The resultant cell suspension was filtered through a nytex mesh, washed twice with RPMI 1640, and resuspended in PBS at $2\times10^7$/ml cells. Immunofluorescence staining and flow cytometry analysis were then performed. The following antibodies were utilized: phycoerythrin conjugate antimouse L3T4 and fluorescein conjugate antimouse Lyt-2 (Becton Dickinson, Mountain View, CA) for a two-colors analysis of CD4- and CD8-positive cells, respectively; fluorescein conjugate anti-CD3-$\varepsilon$ (clone 145-2C11) (Boehringer Mannheim Bioch., Mannheim, Germany) for a single-color analysis; fluorescein conjugate anti-$\alpha\beta$ TCR (H57-597 mAb), kindly provided by L. Jones (NCI, NIH, Bethesda), for a single-color analysis. Phycoerythrin conjugate antihuman CD4 and fluorescein conjugated antihuman CD8 (Becton Dickinson) were used as unrelated controls for background detecting. Staining was performed at 4 °C for 30 min. After treatment, cells were washed twice in PBS containing 0.02% sodium azide and flow cytometry analysis was performed using a FACScan (Becton Dickinson). In two-color analysis, marks were set to indicate quadrant boundaries limiting 99.8% of the background events in the lower left quadrant. In single-color analysis, markers were set to indicate the upper and lower boundaries of CD3/TCR-$\alpha\beta$ lo, int, or hi populations for comparison among treatment groups. The first boundary was obtained by limiting 99.7% of the background events, and the others were set arbitrarily on the basis of the curve profile obtained in control samples and maintained in the experimental samples. Data collection was gated on live thymocytes by forward and side angle scatter, utilized to exclude dead cells, debris, very large nonlymphoid cells, and cell aggregates. Data represent 5000 events.

### Immunocytohistochemistry

Immunostaining on paraffin included thymus sections and freshly isolated thymocytes was performed 3 hr after the last DI-M-$PGE_2$ injection using as primary antibody (diluted 1:100) an affinity-purified monospecific rabbit IgG raised against soluble "tissue transglutaminase" of human red blood cells (kindly furnished by L. Fesus, University Medical School of Decebren, Hungary) in a wet chamber overnight at 4 °C. Biotinylated goat antirabbit IgG was used as a second antibody followed by a preformed avidin-horseradish peroxidase complex (Immunon, Detroit, MI). The reaction was developed using aminoethylcarbazole as chromogen substrate and 0.01% $H_2O_2$. Cells were counterstained in Mayer's hemalum. Endogenous peroxidase activity was blocked by methanol-$H_2O_2$. Isolated thymocytes were obtained as previously described for flow cytometry analysis. After extensive washing in PBS, cells were smeared on slides, then fixed in 2.5% paraformaldehyde and, after immunostaining, counterstained with Mayer's hemalum.

### Tissue Transglutaminase Activity

Thymuses were collected from control or Di-M-$PGE_2$, at various time intervals after $PGE_2$ administration, extensively washed in PBS, and homogenized in 0.1-M Tris-HCl, pH 7.5, containing 0.25-M sucrose, 0.5-mM EDTA, and 1-mM PMSF. Transglutaminase activity was measured by detecting the incorporation of ($^3$H)putrescine into N,N'-dimethylcasein. The incubation mixture contained 150-mM Tris-HCl buffer, pH 8.3, 5-mM $CaCl_2$, 10-mM dithiothreitol, 30-mM NaCl, 2.5-mg N,N'-dimethylcasein/ml, 0.2-mM putrescine, containing 1-mCi ($^3$H)putrescine, and 0.1–0.2-mg protein in a final volume of 0.3 ml. After 20 min of incubation, the mixture was spotted onto Whatman 3 mm filter paper moistened with 20% trichloroacetic acid (TCA). Free ($^3$H)putrescine was eliminated by washing with large volumes of cold 5% TCA containing 0.2-M KCl before counting. tTG activity was calculated as nanomoles of ($^3$H)putrescince incorporated into protein per hour, and was expressed as a percentage from values obtained in mice treated with control diluent.

### ACKNOWLEDGMENTS

We thank L. Fesus (University Medical School of Decebren, Hungary) for the anti-tTG antibody and for helpful discussions; L. Jones (NCI, NIH, Bethesda) for H57-597 mAb; G Febbraro for technical assistance. This work was supported by C.N.R. Spec. Project "Aging" and by Min. Sanità Project AIDS.

*(Received November 4, 1991)*

*(Accepted December 3, 1991)*

### REFERENCES

Arends M., and Wyllie, A.H. (1991). Apoptosis: Mechanisms and role in pathology. Int. Rev. Exp. Pathol. **32:** 223–254.

Betz M., and Fox B.S. (1991). Prostaglandin E2 inhibits production of Th1 lymphokines but not of Th2 lymphokines. J. Immunol. **146:** 108–113.

Boyd R.L., and Hugo P. (1991). Towards an integrated view of thymopoiesis. Immunol. Today **12:** 71–79.

Fesus L., Thomazy V., Autori F., Cerù M.P., Tarcsa E., and Piacentini M. (1989). Apoptotic hepatocytes become insoluble in detergents and chaotropic agents as a result of a transglutaminase action. FEBS Lett. **245:** 150–154.

Fesus L., Thomazy V., and Falus A. (1987). Induction and activation of tissue transglutaminase during programmed cell death. FEBS Lett. **224:** 104–108.

Gallily R., Zeira M., and Stain I. (1985). Thymus-derived macrophages in long-term culture: Release of IL-1, stimulation of MLR and expression of tumoridical activity. Immunology **55:** 165–172.

Goodwin J.S. Ed. (1985). Prostaglandins and immunity (Boston: Martinus Nijhoff).

Homo-Delarche F., Duval D., and Papiernik M. (1985). Prostaglandin production by phagocytic cells of the mouse thymic reticulum in culture and its modulation by indomethacin and corticosteroids. J. Immunol. **135:** 506–512.

Jenkinson E.J., Kingstom R., Smith C.A., Williams G.T., and Owen J.T. (1989). Antigen-induced apoptosis in developing T cells: A mechanism for negative selection of the T cell receptor repertoire. Eur. J. Immunol. **19:** 2175–2177.

Kizaki H., Tadakuma T., Odaka C., Muramatsu J., and Ishimura Y. (1989). Activation of a suicide process of thymocytes through DNA fragmentation by calcium ionophores and phorbol esters. J. Immunol. **143:** 1790–1794.

McConkey D., Orrenius S., and Jondal M. (1990a). Agent that elevate cAMP stimulate DNA fragmentation in thymocytes. J. Immunol. **145:** 1227–1230.

McConkey D., Orrenius S., and Jondal M. (1990b). Cellular signaling in programmed cell death (apoptosis). Immunol. Today **11:** 120–121.

McCormack J.M., Kappler J., Marrak P., and Westcott J.Y. (1991). Production of Prostaglandin E2 and prostacyclin by thymic nurse cells in culture. J. Immunol. **146:** 239–243.

Murphy K.M., Heimberger A.B., and Loh D.J. (1990). Induction by antigen of intrathymic apoptosis of $CD4^+CD8^+TCR^{lo}$ thymocytes in vivo. Science **250:** 1720–1723.

Nakashima M., Mori K., Maeda K., Kishi H., Hirata K., Kawabuchi M., and Watanabe K. (1990). Selective elimination of double-positive immature thymocytes by a thymic epithelial cell line. Eur. J. Immunol. **20:** 47–53.

Nieburgs A.C., Korn J.H., Picciano P., and Cohen S. (1985). The production of regulatory cytokines for thymocyte proliferation by murine thymic epithelium in vitro. Cell. Immunol. **90:** 426–438.

Ohashi P.S., Pircher H., Bulki K., Zinkernagel R.M., and Hengartuer H. (1990). Distinct sequence of negative or positive selection implied by thymocyte T-cell receptor densities. Nature **346:** 858–860.

Ojeda F., Guarda M.I., Maldonado C., and Folch H. (1990). Protein kinase-C involvment in thymocyte apoptosis induced by hydrocortisone. Cell. Immunol. **125:** 535–539.

Piacentini M., Autori F., Dini L., Farace M.G., Ghibelli L., Piredda L., and Fesus L. (1991a). "Tissue" transglutaminase is specifically expressed in neonatal rat liver cells undergoing apoptosis upon epidermal growth factor-stimulation. Cell Tissue Res. **263:** 227–235.

Piancentini M., Fesus L., Farrace M.G., Ghibelli L., Piredda L., and Melino G. (1991b). The expression of "tissue" transglutaminase in two human cancer cell lines is related with the programmed cell death (apoptosis). Eur. J. Cell Biol. **54:** 246–254.

Rinaldi-Garaci C., Favalli C., Del Gobbo V., Garaci E., and Jaffe B.M (1983). Is thymosin action mediated by prostaglandin release. Science **220:** 1163–1164.

Rothemberg E.V. (1990). Death and transfiguration of cortical thymocytes a reconsideration. Immunol. Today **11:** 116–119.

Screpanti I., Morrone S., Meco D., Santoni A., Gulino A., Paolini R., Crisanti A., Mathieson B.J., and Frati L. (1989). Steroid sensitivity of thymocyte subpopulations during intrathymic differentiation. J. Immunol. **142:** 3378–3383.

Shi Y., Sahai B.M., and Green D.R. (1989). Cyclosporin A inhibits activation-induced cell death in T-cell hybridomas and thymocytes. Nature **339:** 625–626.

Shi Y., Bissonete R.P., Parfrey N., Szalay M., Kubo R.T., and Green D. (1991). In vivo administration of monoclonal antibodies to the CD3 T cell receptor complex induces cell death (apoptosis) in immature thymocytes. J. Immunol. **146:** 3340–3346.

Shortman K., and Vremec D., (1991). Different subpopulation of developing thymocytes are associated with adherent (macrophage) or nonadherent (dendritic) thymic rosettes. Develop. Immunol. **1:** 225–235.

Smith C.A., Williams G.T., Kingstom R., Jenkinson E., and Owen J.J. (1989). Antibodies to CD3/T-cell receptor complex induce death by apoptosis in immature T cells in thymic cultures. Nature **337:** 181–184.

Suzuki K., Takaduma T., and Kizaki H. (1991). Modulation of thymocytes apoptosis by isoproteneroI and prostaglandin E2. Cell. Immunol. **134:** 235–240.

Vercammen C., and Ceuppens J.L. (1987). Prostaglandin E2 inhibits T-cell proliferation after crosslinking of the CD3-T complex by directly affecting T cells at an early step of the activation process. Cell. Immunol. **104:** 24–36.

Von Boehmer H. (1991). Positive and negative selection of the T-cell repertoire in vivo. Curr. Opinion Immunol. **2:** 210–215.

Wyllie A.H. (1980). Glucocorticoid-induced thymocyte apoptosis is associated with endogenous endonuclease activation. Nature **284:** 555–556.

Wyllie A.H., Kerr J.F.R., and Currie A.R. (1980). Cell death: The significance of apoptosis. Int. Rev. Cytol. **68:** 251–306.

Wyllie A.H., and Morris R.G. (1982). Hormone-induced cell death. Am. J. Pathol. **109:** 78–87.

Yamada T., and Ohyama H. (1988). Radiation-induced interphase death of rat thymocytes is internally programmed (apoptosis). Int. J. Radiat. Biol. **53:** 65–75.

Zugic J.N. (1991). Phenotypic and functional stages in the intrathymic development. Immunol. Today **12:** 65–69.

